# Geographic variations of childhood asthma hospitalization and outpatient visits and proximity to ambient pollution sources at a U.S.-Canada border crossing

**DOI:** 10.1186/1476-072X-4-14

**Published:** 2005-06-08

**Authors:** Tonny J Oyana, Patrick A Rivers

**Affiliations:** 1 Department of Geography and Environmental Resources, 1000 Faner Drive, MC 4520, Southern Illinois University, Carbondale, IL 62901-4514, USA; 2 Associate Professor and Director. Health Care Management, College of Applied Sciences and Arts, Southern Illinois University, Carbondale, Illinois, USA

## Abstract

**Background:**

Childhood asthma is a significant public health problem in the United States and evidence is accumulating regarding the contribution from traffic and ambient air pollution. This study is a companion piece of a related Buffalo asthma study in adults recently published in the July 2004 issue of *American Journal of Public Health*. This study focuses on children under 18 years of age diagnosed with asthma during a three-year period (2000–2002). In order to determine the effects of particulate air pollution on public health, we conducted an ecologic study of childhood asthma and point-source respirable particulate air pollution in patients diagnosed with asthma (*n *= 6,425). Patients diagnosed with gastroenteritis (n = 5,132) were used as controls.

**Results:**

Although the results of this study show spatial patterns similar to the ones observed in the adult study, a multiple-comparison test shows that EPA-designated focus sites located in Buffalo's east side are statistically (*p *< 0.008) more linked to childhood asthma than sites located elsewhere.

**Conclusion:**

Findings of this study can be useful in geographic targeting and in the design of optimal and preventive measures.

## Introduction

Asthma is a growing public health problem within the United States. It affects approximately 15 million people and results in at least 2 million emergency room visits and more than 5,000 deaths each year [1], with an estimated 6.7% prevalence of childhood asthma [2]. Moreover, there is mounting evidence showing that asthma prevalence in childhood has plateaued over the past two decades [1,3-5]. Surveillance records from the Centers for Disease Control and Prevention (CDC) also show an upward trend within the neighborhood of 1.74 times for the period between 1980 and 1996 [1].

Asthma is a chronic inflammatory disease of the airways which is associated with reversible airway obstructive, hyperresponsiveness to triggers, clinical symptoms of wheezing, chest tightness, or cough and increased mucous production. It is a major respiratory illness among children and disproportionately affects minorities [6]. Most children diagnosed with asthma have mild to moderate symptoms, however, there are those whose symptoms result in numerous visits to the hospital emergency room and multiple hospitalizations.

Asthma remains a major burden within the Buffalo community located near the U.S.-Canada border, and there is growing evidence from past studies [7-20] confirming this statement. For example, several published population-based and health care utilization studies have produced persuasive evidence suggesting a combination of contributing factors to asthma exacerbations and prevalence rates in neighborhoods located in close proximity to major traffic zones in western New York [7-14]. The evidence emerging from these reports lends credence to the hypothesis that traffic-related pollutants play a major role in the worsening of asthma and its development.

At the present time, there is consistency among findings reported by previous studies, and at least four explanations are emerging: (1) Increases in hospitalizations due to asthma over a decade were associated with increased truck traffic in ZIP code areas downwind of the Peace Bridge Complex (PBC) and the major roadways supplying it [15]. Following the World Trade Center tragedy on September 11, 2001, there was a decrease in traffic at the border crossing. As a result, there was an associated decrease in hospitalization rates for respiratory illnesses. This effect was reversed when traffic recovered [13]. The Public Bridge Authority measured a six-fold increase in PM_2.5 _(particulate matter that is 2.5 micrometers or smaller in size) levels during the period of September 11, 2001 due to increased delays caused by more detailed customs inspections [16]. (2) A study that focused on analyzing health effects of the U.S. Environmental Protection Agency (EPA) designated sites [17] provided strong evidence to support the hypothesis that asthma risk increases as distance to these focus sites decreases. (3) Two house-to-house surveys of home environmental factors [18,19], conducted six years apart, suggested that household triggers such as smoking, humidifiers, and age of housing units were associated with increased asthma prevalence. (4) A separate analysis of socioeconomic factors [14,18,20] suggested that asthma prevalence varied by race, gender, and maternal history of asthma among children 4 to 13 and women 18 to 54 years of age. Additionally, a recent study of risk factors for asthma showed that location, gender, age, and race were significant factors even after adjusting for age of housing, pets, molds, animal triggers, and smoking [14].

While findings from previous studies have assisted in the characterization of the magnitude and geographic extent of asthma, none of the studies have focused on childhood asthma in the city of Buffalo and its environs. In this study, we report on hospital visits by children diagnosed with asthma. The rationale for studying childhood asthma stems from the need to understand health care utilization rates among children in the study area. The study takes advantage of readily available automated administrative datasets for childhood asthma patients since 2000 by Kaleida Health Systems.

## Results and discussions

Kaleida asthma and gastroenteritis databases for children contained 6,425 and 5,132 hospitalization records, respectively. Of these records only 89.2% and 89.7% listed a residential address. The geocoding processing yielded over 90% address matching for both databases. The geocoding success was due, in part, to improved data management practices established after previous studies.

Table 1 presents exposure in geographic locations identified at the ZIP code level. The highest percentages of diagnosed childhood asthma were seen in ZIP codes 14215 and 14211, located east of Buffalo, and ZIP codes 14201 and 14213, located west of Buffalo. However, the lowest percentages were seen in ZIP codes located farther away. The highest childhood asthma hospitalization rate per 10,000 population was recorded in ZIP code 14203, however, this was not statistically significant. We observed statistically significant positive associations at the 95% significance level between exposure sites and outcomes in ZIP codes 14212, 14204, 14211, and 14214, but a negative association was found in ZIP codes 14201, 14202, 14225, 14221, and 14224.

**Table 1:** Exposure based on Geographic Locations Identified at the ZIP Code Level: Odds Ratios from a Case-Control Study, 2000–2002

	**Case Patients (n = 5,731)**		**Control patients (n = 4,604)**			
				
Zip Code	% of Diagnosed Asthma Cases	Asthma Hospitalization Rates (per 10 K)	% of Diagnosed Gastroenteritis	Gastroenteritis Hospitalization Rates (per 10 K)	Odds Ratios	95% Confidence Interval (CI)
14203	0.63	356.79	0.48	218.04	1.31	[0.779, 2.218]
14212	7.17	267.51	4.87	145.80	1.51	[1.280, 1.778]**
14204	4.34	248.01	3.52	161.35	1.24	[1.019, 1.518]**
14213	11.22	246.55	12.19	215.11	0.91	[0.807, 1.027]
14201	5.69	239.55	6.93	234.40	0.84	[0.718, 0.988]*
14211	11.85	229.47	8.84	137.55	1.39	[1.220, 1.575]**
14208	4.26	184.75	3.82	133.26	1.12	[0.920, 1.364]
14215	12.84	165.45	12.05	124.76	1.08	[0.956, 1.209]
14209	1.97	132.21	2.09	112.32	0.94	[0.715, 1.240]
14207	4.96	123.55	5.41	108.33	0.91	[0.766, 1.088]
14214	3.35	87.83	2.13	44.83	1.59	[1.252, 2.026]**
14206	2.62	65.25	2.39	47.85	1.10	[0.858, 1.408]
14210	1.41	48.56	1.67	46.16	0.84	[0.613, 1.157]
14202	0.33	45.94	0.63	70.12	0.52	[0.286, 0.952]*
14216	1.80	43.67	2.28	44.52	0.79	[0.595, 1.037]
14222	0.92	36.90	0.83	26.45	1.11	[0.732, 1.682]
14220	1.68	36.16	1.52	26.37	1.11	[0.813, 1.507]
14217	1.20	27.94	1.24	23.08	0.97	[0.679, 1.378]
14218	0.96	27.22	1.22	27.72	0.78	[0.537, 1.146]
14226	1.36	26.55	1.61	25.19	0.84	[0.610, 1.164]
14225	1.61	25.63	2.45	31.48	0.65	[0.490, 0.866]*
14223	0.89	21.17	0.96	18.26	0.93	[0.617, 1.391]
14228	0.68	19.78	0.74	17.25	0.92	[0.578, 1.460]
14227	0.59	13.84	0.93	17.50	0.63	[0.398, 1.005]
14221	0.96	10.69	1.46	13.02	0.65	[0.453, 0.944]*
14224	0.61	8.64	1.15	13.08	0.53	[0.339, 0.822]*
14219	0.16	6.90	0.30	10.73	0.53	[0.224, 1.264]

Note. Two denominators are reported, one is derived from the number of children registered in the Kaleida Health System and the other from population data from the 2000 US Census; and case patients and control patients derived from hospitalization and outpatient visits for asthma (ICD-9 code 493) and gastroenteritis (ICD-9 code 558) from Kaleida database, 2000–2002

** Positive association between exposure and outcome at the 5% significance level

* Negative association between exposure and outcome at the 5% significance level

Table 2 presents spatial analysis results of a case-control study showing odds ratios. This study found that proximity to the EPA-designated toxic emission sites [Odds Ratios (OR) = 1.91, 95% confidence interval (CI) = 1.211–3.011] was associated with statistically significant increased odds of having diagnosed asthma but not of non-respiratory disease. However, we also observed a negative association for multiple emission sites [OR = 0.81, 95% CI = 0.703–0.92], PBC [OR = 0.69, 95% CI = 0.48–0.99], and interstate highway [OR = 0.81, 95% CI = 0.69–0.95].

**Table 2:** Spatial analysis of Case-Control Study Showing Odds Ratios

	Odds Ratios		95% Confidence Interval	
		
Sites	0.5 vs 2 km	1 vs 2 km	0.5 vs 2 km	1 vs 2 km
Peace Bridge Complex	0.69*	0.87	0.48,0.99	0.69,1.09
Air release	0.77	0.87	0.58,1.03	0.70,1.09
Toxic release	1.91**	1.23	1.21,3.01	0.88,1.72
Multiple release	0.80*	0.93	0.70,0.92	0.83,1.05
Interstate 190	0.90	0.81*	0.74,1.08	0.69,0.95
Interstate198 and Route 33	0.96	0.95	0.85,1.09	0.85,1.07
Main St, Bailey Ave, Niagara St, and Seneca St	0.92	0.96	0.82,1.02	0.86,1.08
Delaware Ave	0.85	1.01	0.68,1.07	0.83,1.24

Note. Case patients and control patients derived from hospitalization and outpatient visits for asthma (ICD-9 code 493) and gastroenteritis (ICD-9 code 558) from Kaleida Database, 2000–2002

** Positive association between exposure and outcome at the 5% significance level

* Negative association between exposure and outcome at the 5% significance level

Table 3 summarizes multiple comparison results for seven EPA-designated focus sites using Diggle's model. The seven sites were statistically significant, hence the need to compare them further. The comparison results are ranked in order of adjusted significance level according to Bonferroni's and Holm's correction methods. The test showed that EPA-designated focus sites located in Buffalo's east side showed a closer association to childhood asthma than sites located elsewhere (p < 0.008). EPA-designated focus sites located near the grain factory and major roadways also showed a strong correlation with childhood asthma.

**Table 3:** Multiple-Comparison Test for Model Fittings according to Diggle's model

EPA-Designated Pollution Sites	Original *P*-value	Modified Holm's	Remarks
Nabisco Company	0.00000	0.0043	Located in the east near the grain factory
Truck Parking Lot, Nabisco Company	0.00000	0.0047	Located in the east near the grain factory
Marnap Industries	0.00000	0.0051	Located in the east near the grain factory
Harrison Radiator	0.00063	0.0127	Located in the east near the major roadway
Miken Company	0.00096	0.0169	Located in the west near the major roadway
Peace Bridge Complex	0.00150	0.0253	Located in the west near the major roadway (I-90)
Birge Company	0.03148	0.05	Located in the west near the major roadway

**Figure 2:**
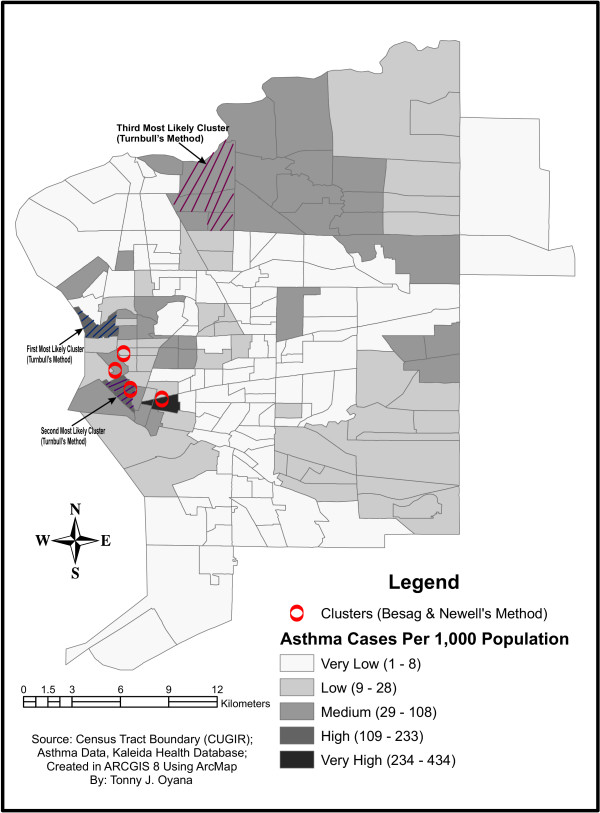
shows spatial clusters of adult asthma. Spatial clusters were detected in Buffalo's west side, parts of the downtown areas, and only 1 geographic region had over 234 asthma cases per 1,000 population.

**Figure 3:**
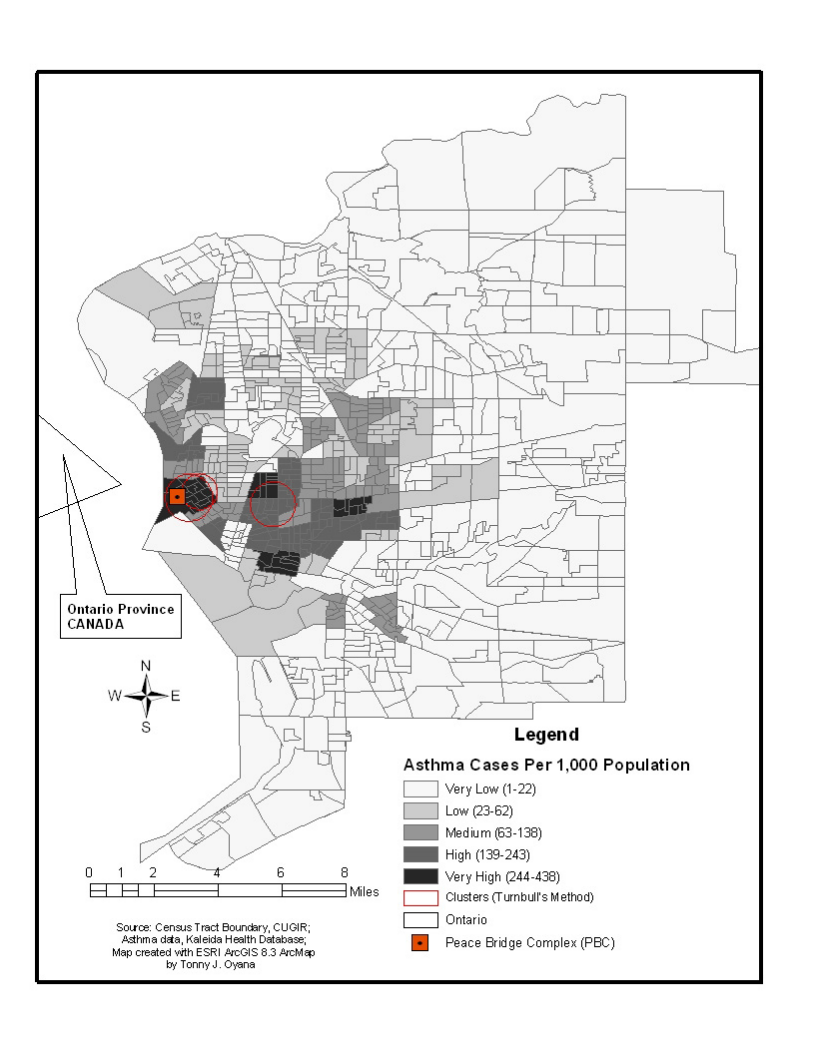
shows spatial clusters of childhood asthma. Spatial clusters were detected in Buffalo's west side, east side, parts of the downtown areas, and 4 geographic regions had over 244 asthma cases per 1,000 population.

Figures 2 and 3 both illustrate spatial clusters of diagnosed asthma in Buffalo neighborhoods. In Figure 2, only one geographic region had over 234 adult asthma cases per 1,000 population [12,17], however, in Figure 3, four geographic regions had over 244 childhood asthma cases per 1,000 population. Similar spatial patterns and distributions of asthma are apparent in both figures, but further analysis revealed that asthma is more prevalent in children than adults.

### Establishing the effects of ambient air pollution on asthma

The need exists for establishing the effects of particulate air pollution on adults and children from the study region. Results of the adult study are already reported in Oyana *et al*. [17].

The adult and children studies were conducted during 1996–2000 and 2000–2002, respectively. The two studies could assist us in elucidating the effects of ambient air pollution in asthma, especially among the children. Based on the outcome measures we should be able to tease out the health effects given that ambient pollution sources being considered have been in operation since the 1980s and transport mechanisms have not showed any significant changes in the past 10 years. Previously, Oyana *et al*. [17] conducted the study involving 3,717 and 4,129 adult asthma and gastroenteritis patients. In the current study, the numbers of child asthma and gastroenteritis patients were 5,731 and 4,604, respectively.

A comparative analysis was conducted both at ZIP code level and at different sites to establish the role of different exposures on asthma in adults and children. At the ZIP code level, a positive association was found between exposure and outcome at a 5% confidence interval in adults in the ZIP codes 14201, 14213, 14207, and 14204. In a similar study for children, the ZIP codes 14212, 14204, 14211, and 14214 showed a positive association.

Similarly, negative association between exposure and outcome at a 5% confidence interval in adults was observed in the ZIP codes 14221, 14214, 14217, 14150, and 14227, whereas in children, it was observed in ZIP codes 14201, 14202, 14225, 14221, and 14224.

Only the ZIP code of 14204 had a positive association in both adults and children, whereas, the ZIP code of 14221 had a negative association. However, there were some differences in both studies. For example, ZIP code 14201 showed a positive association in the adult study, while a negative association was observed for the children's study. Likewise, ZIP code 14214 had a negative association for the adult study, but a negative association was found for the children's study.

In adults, we observed the highest odds ratio of 15.77 at air release sites at 0.5 versus 2 km, however, in children, we found the highest odds ratio of 1.91 in the toxic release site at 0.5 versus 2 km. When comparing the 1 km and 2 km scenario, the adult study showed two positive associations, whereas the child study showed none. The adult study revealed a strong positive relationship for the Interstate 190 roadway for both scenarios; however, in the child study, none of the sites showed a positive relationship in either the 0.5 km versus 2 km or 1 km versus 2 km scenarios. In addition, although there are four sites with positive associations in the adult study, it does not include the toxic release site, which is the only site with positive association in the child study.

Table 3 shows the results of the multiple comparison test using Diggle's method. The data shows that EPA-designated focus sites on the east side of Buffalo are more statistically linked to childhood asthma than those on the west side. Three sites in the east have a *p*-value = 0.0000 and have shown the strongest statistical significance in a multiple comparison study. The two studies reveal that EPA-designated focus sites including the PBC are significant contributors to increasing the number of asthma incidents in the study area.

The spatial clusters of adult asthma seen in Figure 2 are on Buffalo's west side and parts of downtown. However, spatial clusters of childhood asthma (Figure 3) were found not only on Buffalo's west side and parts of downtown, but also on its east side. This could be explained by African-American and Hispanic groups, which comprise a large portion of the population on the Eastside. The adult study reveals that only one geographical area has more than 234 asthma cases per 1,000 population, while the children's study shows that there are four geographical areas with more than 244 asthma cases per 1,000 population. Figures 2 and 3 suggest that children are more susceptible to asthma than adults in the study region, possibly due to incomplete development of the immune system in children. An ongoing study is collecting field measurements of particulate matter concentrations based on sampling sites identified within and outside of the spatial clusters in Figures 2 and 3. Preliminary findings of large variations of PM_2.5 _concentrations were observed in locations of high asthma prevalence and there are consistent with the results of the GIS disease model. The site observations reported in this ongoing study confirms the relevance of identified spatial clusters and lends further credence to their use in GIS modeling.

There are two major observations that can be made regarding both studies: (1) Findings in the children's study are consistent with previous findings in the adult study. Current findings support the hypothesis that traffic levels at the U.S.-Canada border crossing point and specific EPA-designated focus sites are associated with high incidences of asthma and prevalence rates, especially in areas that are located in the west and east of Buffalo. The two studies have further established credible evidence of statistical associations between current traffic levels on major roadways and specific EPA-designated focus sites [18-20]. (2) A huge disease risk and burden exists especially among children. This burden, or risk, is confined not only to children residing on Buffalo's west side but also to those children on the east side. This second observation highlights the need to focus preventive and mitigation measures and efforts on residents living in close proximity to the border crossing point, especially on children.

The policy implication of our study is targeting exposure reduction. This would be justified on the grounds of maximizing public health benefits. Differential distribution of adverse health effects also need to be considered alongside differential distribution of the benefits related to the emission sources.

## Conclusion

This childhood asthma study, as did the adult study, demonstrates the effects of ambient pollution sources on individuals with asthma and suggests these sources are the contributing factors both in the west and east of the study area. Identification of asthma clusters associated with different sources may provide insights into how mixtures of pollutants interact and lead to development of asthma in susceptible individuals. These findings provide a basis for a better understanding of outdoor environmental factors that might be related to the spatial distribution of asthma prevalence and morbidity in this Buffalo community.

## Materials and Methods

### Study area and Population

**Figure 1:**
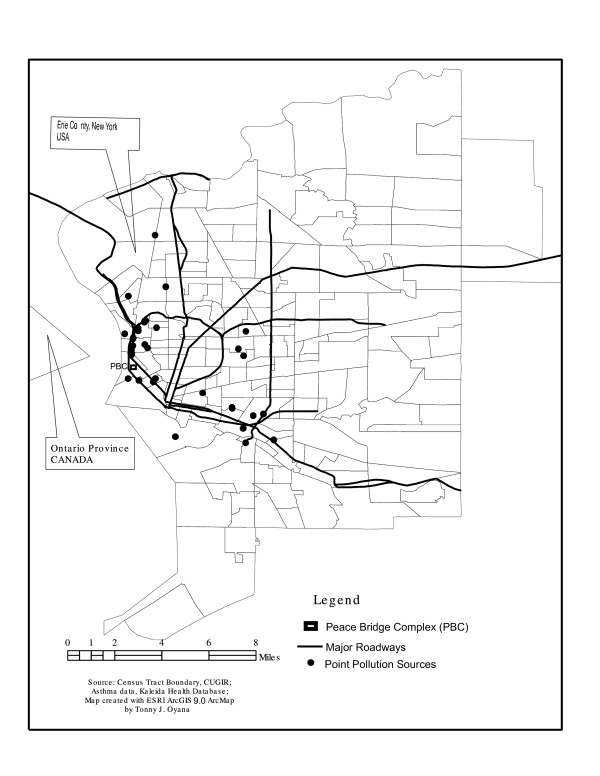
shows the study area, major roadways, EPA-designated point pollution sources, and the location of the Peace Bridge Complex (PBC).

The study area, as shown in Figure 1, is the second-largest city in New York State, with a population near 600,000. Most of these people reside in the city of Buffalo and its surrounding areas. The study area, which consists of approximately 27% children between age 1 and 18 years of age, serves as the main traffic corridor between the United States and Canada. It has a rich history of industrial activity and is also an emerging international trade corridor for the United States, Mexico, and Canada under the North American Free Trade Agreement (NAFTA), with a mandate to promote trade among the member states. It is one of the busiest trade corridors, with increased truck traffic traveling through densely populated residential areas and increasing the potential for the release of pollutants such as nitrogen dioxides and particulate matter that have an adverse effect on human health. In fact, a number of studies have associated these pollutants with the exacerbation of respiratory diseases [21-28]. The study area also includes both industrialized and non-industrialized neighborhoods in order to provide access to diverse populations.

### Data sources

Three data sources were analyzed in this study: (1) hospitalization and outpatient visits for asthma (ICD-9 code 493) and gastroenteritis (ICD-9 code 558); and (2) EPA-designated focus sites.

1. Hospitalization and Outpatient Visits for Asthma and Gastroenteritis for children age 1 to 18 years, encompassing admissions from December 2000 to December 2002 available from Kaleida Health System, which includes Buffalo General Hospital, Millard Fillmore Hospitals (Gates and Suburban), DeGraff Memorial Hospital, and Children's Hospital of Buffalo. Each record in the database was assigned a unique identifier ensure patient confidentiality. Data on child age, residential addresses, and insurance status were derived from this source. Home locations of patients were the basis for measuring possible residential exposure to known pollution sites.

Gastroenteritis patients were designated as the non-respiratory control group because it has no connection to ambient air quality and in some ways this "adjusts" for other aspects of a person that lead to access to care/hospitalization such as socioeconomic status (SES). Overall, processing and management of the datasets were of high quality as a result of previous knowledge of adult asthma [12] and also because of strong experience in geocoding residential addresses in the study area. Moreover, the Kaleida Health System is western New York's largest healthcare provider with a market share of 52% and serves approximately 80% of the total population in the study area.

2. EPA-designated focus sites used for this study are similar to those reported in Oyana and Lwebuga-Mukasa [12] and Oyana *et al*. [17]. The childhood asthma study, like the adult study, searched for potential spatial relationships between these sites and home locations of children who were diagnosed with asthma. We calculated distances between residential locations for each patient to each EPA-designated focus site.

Additional geographic information, such as the US Census data on population, major roadways, boundary files, was obtained from Cornell University Geospatial Information Repository (CUGIR) and the Federal Geographic Data Committee (FGDC) Clearinghouse Node for New York State.

### Data analysis

Statistical and spatial techniques were used to study spatial location of case patients and control patients in relation to their proximity to different pollution sources. The details of these techniques have been discussed in earlier reports [17] and will only be presented briefly. In general, the data are explored in a number of ways.

1. Description of ZIP code level differences in rates of asthma and gastroenteritis hospitalization and outpatient visits

2. Case control analysis of proximity to important sources of ambient air pollutants

3. Cluster identification (Diggle's method)

Data processing, GIS mapping, and analysis were conducted in ESRI ArcGIS 9.0 (ESRI, Inc., Redlands, California) and Microsoft Excel (Microsoft, Inc., Seattle, Washington). ClusterSeer 2.03 (TerraSeer, Inc., Ann Arbor, Michigan) was used to implement cluster analysis and Diggle's model. Final map production was completed using Corel Draw 11 (Corel Corporation, Ltd., Ottawa, Ontario).

Diggle's method is a focused cluster detection approach appropriate for handling spatial data at the individual level [29,30]. The method compares the spatial pattern of case locations with the spatial pattern of control locations, for instance using a more common "control" disease. The control acts as a null model of no clustering and normally reflects the spatial pattern of the population-at-risk. The test is based upon maximizing the likelihood of the sample of cases and controls, which in turn is based on an exponential decline in risk as the squared distance from the source increases.

It was assumed that those who lived within 1 km of the emission sites and busily traveled roadways were exposed to vehicle exhaust fumes and pollutants from suspected sources of pollution, and those living further away more than 2 km were assumed to be unexposed. Rijnders *et al*. [31] recommend that variables such as degree of urbanization, traffic density, and distance to a nearby highway or any potential pollution source can be used to estimate exposure to traffic-related air pollution. Milligan *et al*. [32] also used distance of more than 2 km in their study to estimate exposure due to traffic-related air pollution. Similarly, the use of data on location of home with respect to roads and of data on traffic density on those roads resulted in observations of significant relationships with specific respiratory hospital admission rates in Toronto [33], with childhood asthma hospitalization rates in Erie County, New York [11], and with childhood asthma medical care visits in San Diego County, California [34].

Epidemiological methods based on odds ratios (OR) and 95 percent confidence intervals were used to compute the spatial risk relationships between cases and controls (using a significance level of ρ ≤ 0.05). A 2 by 2 table analysis was conducted to demonstrate the relationship between two dichotomous or binary variables (exposed and unexposed groups). The variable we are measuring for the 2 × 2 table is the percentage of children who not only were clinically diagnosed with asthma, but also who reside near major roadways. The other variable represents those who reside farther away. We were interested in this type of analysis because previous studies [31-37] have shown that there is a significant risk among patients living near major traffic zones. The emerging evidence suggests that major traffic zones influence the patients' susceptibility to respiratory illnesses, especially among persons with asthma. Moreover, by measuring the residential locations of patients thought to be exposed and comparing it with those living farther away, we were able to quantify the odds of the disease burden among the study population which could be attributed to the exposure or non-exposure sources.

### Limitations of Current Work

Nevertheless, it is likely that we were not able to fully control for the effect of all confounders. Other potential confounders that we were not able to measure include duration of residence, comorbidity, smoking, and exposure to other pollutants in vehicle emissions. Previous studies suggest that control for duration of residence has little influence on effect estimates [33-35], possibly because of an acute effect of exposure. We considered the use of consumer purchasing data to control for area-level smoking, but available data were of questionable validity. Given the similar dispersion characteristics of PM_2.5 _and other pollutants in vehicle emissions (e.g., NO_2_), some of the observed effect may be caused by exposure to other pollutants. Finally, this study does not take into account other possible factors for the prevalence of asthma, such as exposure to indoor pollutants and occupational exposures.

## Protection of Human Subjects

All research reported in this article was approved by the Southern Illinois Unversity Carbondale and the University at Buffalo Human Investigation Review Board in accordance with national and institutional guidelines for the protection of human subjects.

## Authors' contributions

Dr. Tonny J. Oyana designed and developed the study's spatial and GIS approach. He also participated in data processing, geocoding, data analysis, and GIS modeling, and he wrote the manuscript. Dr. Patrick A. Rivers advised on healthcare issues and participated in the final editing and reviewing of the manuscript.
